# Supplementary figures and images for: Hybrid PBL and Pure PBL: Which one is more effective in developing clinical reasoning skills for general medicine clerkship?—A mixed-method study
Source: PLoS One. 2023 Jan 23;18(1):e0279554. doi: 10.1371/journal.pone.0279554 (PMC9870130; doi:10.1371/journal.pone.0279554)

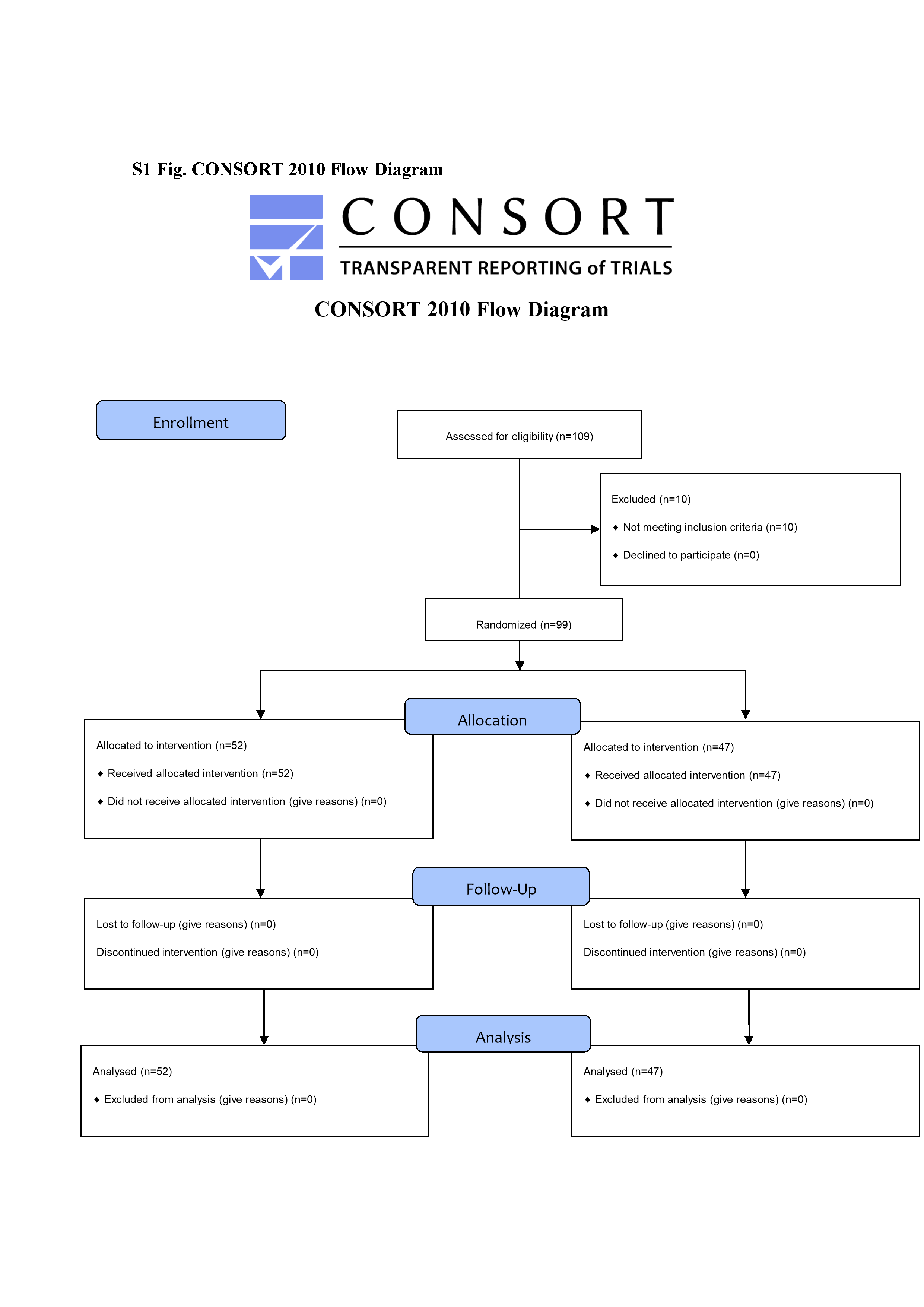

Supplement: S1 Fig — (TIF) [file pone.0279554.s001.tif]
