# Supplementary material for: Hybrid PBL and Pure PBL: Which one is more effective in developing clinical reasoning skills for general medicine clerkship?—A mixed-method study
Source: PLoS One. 2023 Jan 23;18(1):e0279554. doi: 10.1371/journal.pone.0279554 (PMC9870130; doi:10.1371/journal.pone.0279554)
Supplement: S1 Table — (PDF) [file pone.0279554.s002.pdf]

S1 Table. The outline of the PBL flow

Session 1

(1) Self-introduction and role assignment
-Determination of moderator and board members
(2) Presentation of patient replication video 1 and distribution of paper material 1
-Discovering clues for discussion
(3) Discussion 1
-Creation of initial problem list
-Generation of disease hypothesis (concept mapping)
(4) Presentation of patient replication video 2
- Discovering clues for discussion
(5) Discussion 2
-Discovering clues for discussion
(6) Physical examination
-Verbally review the physical examination required for medical students
(7) Organizing lists of differential diseases and conditions
(8) Organizing concept mapping

(9) Setting learning goals

(10) Review of Session 1

Session 2

(1) Review of Session 1

(2) Distribution of paper material 2-4 and discussion
-Distribution and interpretation of test results
-Distribution and interpretation of imaging findings
(3) Reorganizing lists of differential diseases and conditions
(4) Reorganizing concept mapping

(5) Resetting learning goals

(6) Distribution of special tests and treatment courses

(7) Review of Session 2
