# Supplementary material for: Hybrid PBL and Pure PBL: Which one is more effective in developing clinical reasoning skills for general medicine clerkship?—A mixed-method study
Source: PLoS One. 2023 Jan 23;18(1):e0279554. doi: 10.1371/journal.pone.0279554 (PMC9870130; doi:10.1371/journal.pone.0279554)
Supplement: S2 Table — (PDF) [file pone.0279554.s003.pdf]

S2 Table. The questionnaire of the Fifth-year medical students' perceived competence in PBL, satisfaction with sessions, and self-evaluation of competency in clinical reasoning about hybrid- and pure-PBL, Chiba University Hospital (N=99)

Questionnaire (7-point Likert scale)
1. Development of knowledge structure for use in clinical contexts
2. Development of an effective clinical reasoning process
3. Development of effective self-directed learning skills
4. Provision of encouragement and motivation for learning
5. Development of team skills
6. Satisfaction with sessions
7. Recalling appropriate history, physical examination, and tests on clinical hypothesis generation
8. Recalling appropriate differential diagnosis from patient's chief complaint
9. Verbalizing points that fit/don't fit the recalled differential diagnosis appropriately
10. Verbalizing and reflecting appropriately on own mistakes
11. Selecting keywords from the whole aspect of the patient
12. Examining the patient while visualizing his/her daily life
13. Considering biological, psychological, and social perspectives
14. Practicing the appropriate clinical reasoning process
