# Supplementary material for: Hybrid PBL and Pure PBL: Which one is more effective in developing clinical reasoning skills for general medicine clerkship?—A mixed-method study
Source: PLoS One. 2023 Jan 23;18(1):e0279554. doi: 10.1371/journal.pone.0279554 (PMC9870130; doi:10.1371/journal.pone.0279554)
Supplement: S3 Table — (PDF) [file pone.0279554.s004.pdf]

S3 Table. Interview guidelines

(1) Introductory conversation	Thank you, the purpose of this study, informed consent, and permission to record on audiotape.
(2) Lead the interview using the following questions<br>(note: care should be taken to respect the flow of discussion)	(a) Please introduce yourself.<br>(b) Think of the advantages of Hybrid PBL. Why do you think they are advantages?
(3) Conclusion	Would anybody like to say anything else about this topic?
