# Supplementary material for: Hybrid PBL and Pure PBL: Which one is more effective in developing clinical reasoning skills for general medicine clerkship?—A mixed-method study
Source: PLoS One. 2023 Jan 23;18(1):e0279554. doi: 10.1371/journal.pone.0279554 (PMC9870130; doi:10.1371/journal.pone.0279554)
Supplement: Attachment — Submitted filename: PLOS ONE REVIEW.docx [file pone.0279554.s005.docx]

PLOS ONE REVIEW: Hybrid PBL and Pure PBL: Which one is more effective in developing clinical reasoning skills for general medicine clerkship? - A mixed-methods randomized control trial

# General Comments:

The authors aimed to test whether a hybrid PBL approach that adds a lecture session to pure PBL, enhanced the medical students’ clinical reasoning competencies compared to pure PBL. This is an important and novel question.

Their approach to the problem is particularly interesting since they have adopted a combination of a randomized controlled trial design with a qualitative focus group to assess the in-depth experience of the learners. Moreover, the authors have assessed the metacognitive aspects of clinical reasoning among their outcomes. The video supplement to the PBL sessions that included the patient’s lifestyle also grabbed my attention.

However, there are some major concerns regarding this piece of research and the way it’s presented that need to be addressed before considering the manuscript for publication in PLOS One.

- The manuscript is lengthy and this has buried its interesting and important aspects. It can be summarized to at least half its current length without sacrificing important points.
- There are major concerns about the clarity of some parts of the text.
- The authors should clarify some methodological aspects detailed below but namely
  - their approach to randomization has not been described,
  - the high number of outcomes that may increase the likelihood of a type I error, and how the authors have adjusted for the effects of baseline difference,
  - And that the actual sample size was smaller than the calculated sample size and this may pose questions regarding the reliability of the negative findings.

# Introduction:

The introduction is hard to follow and contains unnecessary explanations. I suggest summarizing it by only including a few sentences on the brief description of clinical reasoning and its significance, the components of competency in clinical reasoning, the role of PBL, and its deficits in clinical reasoning development in about two paragraphs. Followed by a third paragraph on the definition of hybrid PBL and how it can solve current problems concerning directly relevant research.

- The term “zone of proximal development” on line 99 is unclear and confusing to me. I guess the authors mean that the learners’ baseline knowledge and past experiences should be taken into account when designing a PBL session.
- There are several paragraphs in the introduction that seem redundant and only bury the main message in a lengthy discussion of unnecessary information.

# Methods:

The RCT methodology is an appropriate method for the authors’ research question. However, some issues need to be addressed. The main concerns are about the randomization methods and the composition of the manuscript.

- The presentation of the methods section can be improved so that it is easier to follow. I suggest using subsections for the “participants and trial design” section: The design: where you describe the study briefly; The Participants and setting: where you describe your participants and their background including the details of the medical education system at your institution with special attention to PBL training.
- Lines 156-159 describe the randomization process. There are 3 points here:
  1. 1. Please include a separate subsection for randomization per the CONSORT statement.
  2. 2. The randomization and blinding have not been described in enough detail. I gathered that the design is a cluster randomized trial where the groups of students were randomized rather than individual students.
  3. 3. Moreover, please clarify how you ensured equal numbers in the groups. Simple randomization does not create equal numbers in the two groups. Did you use blocking?
  4. I suggest including all the details about randomization under a separate subsection per the CONSORT checklist. This would improve your manuscript readability and clarity.
- In the intervention subsection, there is confusion over what the authors exactly did in contrast to what they should or could have done. I suggest that the authors describe what they did in this study instead of what a PBL session generally looks like or going back to the setting they conducted the study in. Examples: Lines 160-169.
- Line 180: Did you do a sequential randomization procedure for this?
  1. Was randomization to the cases after randomization to the pure PBL vs Hybrid PBL?
  2. Since the difficulty level of the cases varied and randomization cannot balance the two groups based on 5 cases, was the between-group balance of cases controlled by any means? A better approach would probably be to ensure equal assignment of cases in the study groups rather than randomization.
  3. If the groups were randomized, how did you conceal the random sequence?
- I suggest organizing the intervention into clear paragraphs describing the tutor selection and training, the instructional design, etc. in clearly distinct paragraphs to improve readability.
- I suggest the following order for your presentation to improve the flow:
  1. The general format of the intervention: case-based discussion led by a tutor, the difference between the two study arms.
  2. Selection and training of the tutors: who were the tutors? How did you select/ train them?
  3. The cases and how they were assigned to the groups.
  4. I would also like to know how the cases were developed and standardized for training purposes.
- Outcome measures: Line 193: The term target achievement rate is somehow unclear to me. The authors need to describe it before using it as a heading. I suggest using a more clear and common term instead. I suggest “students’ perceived competence in clinical reasoning”.
- The subsection was hard to follow probably due to lack of coherence within and between paragraphs. I suggest organizing this section to improve readability. One possible sequence would be: the construct, the tools to measure that construct and their reliability, and the way the tools were used in this study.
- Lines 231-233: I do not think this part adds much or helps the reader of your article understand or learn something. The authors may want to omit this. An alternative to this would be a brief statement on why the authors chose KFP and not other assessment methods of clinical reasoning.
- Lines: 237-238: Using the same disease in the KFP as discussed in the PBL session rises an issue: the KFP test may test the gained knowledge on the case during the PBL session in contrast to competence in clinical reasoning.
- Sample size: the authors have used a smaller number of participants than their calculated sample size. This may have increased the type 2 error. I suggest clarifying this with possible technical reasons why the planned number of participants could not be recruited.
- Data analysis: Since the groups were not similar at baseline, I suggest using techniques to adjust for baseline differences.
- Why did the authors only hold the focus groups among the hybrid groups? It occurred to me that the focus group sessions could have a separate effect on students’ competence in clinical reasoning via reflection or simply increased interaction with the tutors. Also, this may introduce bias as a co-intervention in the hybrid group.

# Results:

- Lines 317-318: I don’t understand how a random assignment can compensate the problem. I would appreciate if the authors clarify this.
- 323-325: Statistical testing for baseline difference is discouraged by most methodologists and per the CONSORT statement. A table presenting the main participant characteristics at baseline with means/SD or number/% in each group is a suitable alternative.
- Most of the results section text contains repetition of the material in the tables. Instead, the authors had better calculate effect sizes and confidence intervals for each of main study outcomes and present that in the text. This would bold out the main results and improve readability.

# Discussion:

- Using item numbers in the text as in lines 435-439 makes the text hard to follow and to understand the concepts presented. Instead, I suggest using short words that refer to the item concepts rather than numbers that require the reader to go back to check your tables several times.
- One issue with discussing individual items in your study is that the comparison of too many items without adjustment for p-value may increase the type 1 error.
- I suggest that the authors summarize and re-arrange their manuscript so that it contains shorter paragraphs. This would greatly increase the reader engagement.
- Line 492-502: Did the lecture or the focus group decrease the students’ ability to reflect? This is an important finding to discuss.

# Limitations:

- First: I suggest simplifying this paragraph. eg: Although the study was randomized the study arms deferred at baseline in regards to….
- Mention the effects of co-intervention from the focus groups that may have introduced bias and what it means in the context of your findings. Since the hybrid PBL group scored lower in reflection and verbalization items after the intervention, could this be an adverse impact of the focus group or does it mean that the hybrid PBL approach decreases the students’ reflective thinking even in spite of the possible positive effects of the focus group on reflection?

# Conclusion:

- Please include an overview of the main study conclusion in response to your primary question.
