# Supplementary material for: Hybrid PBL and Pure PBL: Which one is more effective in developing clinical reasoning skills for general medicine clerkship?—A mixed-method study
Source: PLoS One. 2023 Jan 23;18(1):e0279554. doi: 10.1371/journal.pone.0279554 (PMC9870130; doi:10.1371/journal.pone.0279554)
Supplement: Attachment — Submitted filename: Response to Reviewers_Ishizuka.docx [file pone.0279554.s007.docx]

October 29, 2022

Dr. Somayeh Delavari

**Title:** Hybrid PBL and Pure PBL: Which one is more effective in developing clinical reasoning skills for general medicine clerkship? - A mixed-methods randomized control trial

【*Revised title:* Hybrid PBL and Pure PBL: Which one is more effective in developing clinical reasoning skills for general medicine clerkship? - A mixed-methods cluster randomized controlled trial】

**Reference number:** PONE-D-22-20122.R1

Dear Dr. Somayeh Delavari,

Thank you for your e-mail of September 29, 2022, regarding our manuscript, **“Hybrid PBL and Pure PBL: Which one is more effective in developing clinical reasoning skills for general medicine clerkship? - A mixed-methods randomized control trial”**, and for the valuable comments of the reviewers. In order to reflect the design of the study accurately, we changed the title to **“Hybrid PBL and Pure PBL: Which one is more effective in developing clinical reasoning skills for general medicine clerkship? - A mixed-methods cluster randomized controlled trial”**. I have attached our revised manuscript, as well as a point-by-point response to the reviewers’ comments.

We hope that the revised manuscript contains suitable responses to the comments, and we think that it has been significantly improved over the previous submission. We trust that our manuscript is now suitable for publication in PLOS ONE.

Thank you in advance for your kind consideration of our work.

Sincerely yours,

Kosuke Ishizuka, MD, PhD

Department of General Medicine, Chiba University Hospital

1-8-1, Inohana, Chuo-ku, Chiba-city, Chiba, Japan

Tel. +81-43-222-7171 (Ext. 6438); +81-43-224-4758 (Direct line)

Fax. +81-43-224-4758

E-Mail: e103007c@yokohama-cu.ac.jp

**RESPONSES TO REVIEWER 1:**

We wish to express our appreciation to the reviewer for insightful comments that have helped us to improve our paper. We hope that the revised manuscript contains suitable responses to the comments, and we think that it has been significantly improved over the previous submission. We trust that our manuscript is now suitable for publication in PLOS ONE.

## **NOTE**

We highlighted changes of significant issues in yellow (please see “Revised Manuscript with Track Changes”).

**Comments:**

I appreciate the authors for their considerable efforts in revising their paper. It has tremendously improved. However, there are a few remaining concerns that should be addressed before the manuscript is suitable for publication in PONE.

**Response:**

Thank you for your comments.

Per your comments and amendments, we have revised and summarized our manuscript without sacrificing any important points.

We have also answered each of the questions you posed in the revised manuscript.

1) The most significant point is that the manuscript, although reduced in size, is still lengthy and this can affect readability. I suggest summarizing some data presentations in tables instead of presenting every detail in the texts, not repeating methods in the results section, and getting help from a professional editor to cut redundancies. The limitations section is specifically too long for its messages and can be summarized.

**Response:**

Per your suggestion, we have further summarized the indicated sections without compromising the intended meaning. We have also obtained help from a professional editor to further cut any redundancies. We reduced the number of words in the text from 4913 words in the previous manuscript to 4243 words in the revised manuscript. Please find a certificate of proofreading in English attached.

Other reviewer has pointed out that we should specify the steps of qualitative research with numbers. Therefore, we added this point as Table 1 so as not to avoid increasing the number of words.

**Changes:**

- **Text.** (Text; lines 64 to 497.)
- **Table 1.** (Methods; line 283.)
- **Participant characteristics.** (Results; lines 305 to 310.)
- **Table 2.** (Results; line 312.)
- **1) Students’ perceived competence in PBL and satisfaction with sessions.** (Results; lines 314 to 324.)
- **2) Self-evaluation of competency in clinical reasoning.** (Results; lines 326 to 383.)
- **Content analysis.** (Results; lines 385 to 401.)
- **Limitations.** (Limitations; lines 459 to 488.)

2) Regarding the randomization procedure, I still find it confusing. I assume that the authors have done cluster randomization. If that is the case, this should be edited in the paper title. Moreover, the authors explanation brings about confusions regarding allocation concealment in contrast to blinding which are two distinct matters in an RCT design. I suggest getting help from their RCT methodologist on how they can best present their methods.
3) Regarding randomization, was there a random sequence list for inclusion of students? If so, how was it kept from the researchers figuring out the sequence (allocation concealment)?

**Response:**

Thank you for pointing it out.

To clarify any possible confusion, we conducted a cluster randomized controlled trial in this study, not block randomized controlled trial. We have thus changed the paper title to **“Hybrid PBL and Pure PBL: Which one is more effective in developing clinical reasoning skills for general medicine clerkship? - A mixed-methods cluster randomized controlled trial”** to better reflect this.

We got help from methodologist and revised our manuscript in order to clarify our methods. Regarding randomization, there was not a random sequence list for inclusion of students. The study included 109 fifth-year medical students at the Chiba University School of Medicine who participated in a clinical clerkship at our department from January 2020 to October 2021. This study was embedded into a clinical clerkship rotation in the department; thus, the participants were not sampled randomly. In the clinical clerkship, 5-6 medical students per group were rotated through the department every two weeks. A total of 20 groups were rotated during the study period. They were allocated to the intervention or control groups equally through cluster randomization using Microsoft Excel (Microsoft Co., Redmond, WA, USA). The allocation was not blinded to the medical students or the faculty. We have added this to our manuscript.

In addition, we have also added to the limitation section the possibility of bias using this method. Specifically, subjective bias may have affected the results. In addition, the assignment of these groups by the university before the start of this study might also have been biased, which may have affected the study results.

**Changes:**

- Title.
- This study was embedded into a clinical clerkship rotation in the department; thus, the participants were not sampled randomly. (Methods; lines 118 to 120.)
- **Randomization**

The 20 eligible groups were allocated to the intervention or control groups in an equal number of groups. The groups were allocated through cluster randomization using Microsoft Excel (Microsoft Co., Redmond, WA, USA). The allocation was not blinded to the medical students or the faculty. (Methods; lines 219 to 223.)

- Sixth, the allocation to the two groups could not be blinded to the participants and the faculty members, and subjective bias may have affected the results. Furthermore, the assignment of these groups by the university before the start of this study might also have been biased, which may have affected the study results. (Limitations; lines 485 to 488.)

4) I suggest that a statistician confirms that the un-adjusted p-values used by the authors are appropriate given multiple comparisons.

**Response:**

Along with your recommendation, we asked the statistician to perform the statistical analysis for check and confirmed that the un-adjusted p-values we used are appropriate given multiple comparisons.

We performed a two-way repeated measure analysis of variance (ANOVA), where the within-subjects factor was the time of implementation and the between-subjects factor was the method of PBL because there are two factors. In this statistical analysis, it should be described as a two-way repeated measure ANOVA exactly, which we have revised accordingly.

Additional statistical analysis of the normal distribution showed that it did not follow a normal distribution. Therefore, we added to the limitation section the possibility of alpha error in two-way ANOVA.

**Changes:**

- In two-way repeated measure analysis of variance, self-evaluation of competency in clinical reasoning was significantly improved in the intervention group in "recalling appropriate differential diagnosis from patient's chief complaint" (F(1,97)=5.295, p=0.024) and "practicing the appropriate clinical reasoning process" (F(1,97)=4.016, p=0.038). (Abstract; lines 46 to 50.)
- In addition, to examine whether the self-evaluation of competency in clinical reasoning changed between the intervention and control groups, we conducted a two-way repeated measure analysis of variance (ANOVA), where the within-subjects factor was the time of implementation, and the between-subjects factor was the method of PBL. (Methods; lines 234 to 238.)
- In addition, an alpha error may have occurred in the statistical analysis of the two-way ANOVA because it did not follow a normal distribution. (Limitations; lines 464 to 466.)

5) The authors say they have designed their study based on the CONSORT statement. The CONSORT statement is intended to improve the reporting quality and is not about designing your study.

**Response:**

As you have indicated, we have revised the description of CONSORT statement as follows:

**Changes:**

- To improve the reporting quality, we conducted a cluster randomized controlled trial based on the CONSORT 2010 statement (S1 Fig) [12]. (Methods; lines 106 to 108.)

We thank the reviewer for such pertinent comments. We hope that the revised manuscript contains suitable responses to the comments, and we think that it has been significantly improved compared to the previous submission. We trust that our manuscript is now suitable for publication in PLOS ONE.

**RESPONSES TO REVIEWER 2:**

We wish to express our appreciation to the reviewer for insightful comments that have helped us to improve our paper. We hope that the revised manuscript contains suitable responses to the comments, and we think that it has been significantly improved over the previous submission. We trust that our manuscript is now suitable for publication in PLOS ONE.

## **NOTE**

We highlighted changes of significant issues in yellow (please see “Revised Manuscript with Track Changes”).

**Comments:**

The steps of qualitative research are not well explained. Please specify the steps of qualitative research with numbers.

**Response:**

Thank you for pointing it out.

Along with your comments, we clarified the steps of qualitative research with numbers.

Other reviewer has pointed out that we should reduce the number of text word count. Therefore, we added this point as Table 1 so as not to avoid increasing the number of words.

**Changes:**

- For the qualitative research, content analysis was used to analyze the themes (Table 1) [22, 23]. (Methods; lines 270 to 271.)
- **Table 1.** (Methods; line 283.)

We thank the reviewer for such pertinent comments. We hope that the revised manuscript contains suitable responses to the comments, and we think that it has been significantly improved compared to the previous submission. We trust that our manuscript is now suitable for publication in PLOS ONE.

**RESPONSES TO REVIEWER 5:**

We wish to express our appreciation to the reviewer for insightful comments that have helped us to improve our paper. We hope that the revised manuscript contains suitable responses to the comments, and we think that it has been significantly improved over the previous submission. We trust that our manuscript is now suitable for publication in PLOS ONE.

## **NOTE**

We highlighted changes of significant issues in yellow (please see “Revised Manuscript with Track Changes”).

**Comments:**

In this manuscript, the block randomization method was used. According to the type of study, which is an RCT, this method needs to be explained more, such as how many blocks are determined and how permutation was done.

**Response:**

Thank you for your comments.

To clarify any possible confusion, we conducted a cluster randomized controlled trial in this study, not a block randomized controlled trial. We have thus changed the paper title to **“Hybrid PBL and Pure PBL: Which one is more effective in developing clinical reasoning skills for general medicine clerkship? - A mixed-methods cluster randomized controlled trial”** to better reflect this.

We got help from methodologist and revised our manuscript in order to clarify our methods. The study included 109 fifth-year medical students at the Chiba University School of Medicine who participated in a clinical clerkship at our department from January 2020 to October 2021. This study was embedded into a clinical clerkship rotation in the department; thus, the participants were not sampled randomly. In the clinical clerkship, 5-6 medical students per group were rotated through the department every two weeks. A total of 20 groups were rotated during the study period. They were allocated to the intervention or control groups equally through cluster randomization using Microsoft Excel (Microsoft Co., Redmond, WA, USA). The allocation was not blinded to the medical students or the faculty. We have added this to our manuscript.

In addition, along with the other reviewer comments, we have also added to the limitation section the possibility of bias using this method.

**Changes:**

- Title.
- This study was embedded into a clinical clerkship rotation in the department; thus, the participants were not sampled randomly. (Methods; lines 118 to 120.)
- **Randomization**

The 20 eligible groups were allocated to the intervention or control groups in an equal number of groups. The groups were allocated through cluster randomization using Microsoft Excel (Microsoft Co., Redmond, WA, USA). The allocation was not blinded to the medical students or the faculty. (Methods; lines 219 to 223.)

- Sixth, the allocation to the two groups could not be blinded to the participants and the faculty members, and subjective bias may have affected the results. Furthermore, the assignment of these groups by the university before the start of this study might also have been biased, which may have affected the study results. (Limitations; lines 485 to 488.)

We thank the reviewer for such pertinent comments. We hope that the revised manuscript contains suitable responses to the comments, and we think that it has been significantly improved compared to the previous submission. We trust that our manuscript is now suitable for publication in PLOS ONE.
